# Interleukin-1 receptor antagonist haplotype associated with prostate cancer risk

**DOI:** 10.1038/sj.bjc.6602729

**Published:** 2005-08-02

**Authors:** F Lindmark, S L Zheng, F Wiklund, K A Bälter, J Sun, B Chang, M Hedelin, J Clark, J-E Johansson, D A Meyers, H-O Adami, W Isaacs, H Grönberg, J Xu

**Affiliations:** 1 Department of Radiation Sciences/Oncology, Umeå University, Umeå S-901 87, Sweden; 2 Center for Human Genomics, Wake Forest University School of Medicine, Winston-Salem, NC, USA; 3 Department of Medical Epidemiology and Biostatistics, Karolinska Institutet, Stockholm, Sweden; 4 Department of Urology, Johns Hopkins Medical Institutions, Baltimore, MD, USA; 5 Department of Urology and Clinical Medicine, Örebro University Hospital, Sweden and Regional Oncological Center, University Hospital, Uppsala, Sweden

**Keywords:** prostate cancer, inflammation, *IL1-RN*, association, SNPs

## Abstract

IL1-RN is an important anti-inflammatory cytokine that modulate the inflammation response by binding to IL1 receptors, and as a consequence inhibits the action of proinflammatory cytokines IL1*α* and IL1*β*. In this study, we hypothesise that sequence variants in the *IL1-RN* gene are associated with prostate cancer risk. The study population, a population-based case–control study in Sweden, consisted of 1383 prostate cancer case patients and 779 control subjects. We first selected 18 sequence variants covering the *IL1-RN* gene and genotyped these single-nucleotide polymorphisms (SNPs) in 96 control subjects. Gene-specific haplotypes of *IL1-RN* were constructed and four haplotype-tagging single-nucleotide polymorphisms (htSNPs) were identified (rs878972, rs315934, rs3087263 and rs315951) that could uniquely describe >95% of the haplotypes. All study subjects were genotyped for the four htSNPs. No significant difference in genotype frequencies between cases and controls were observed for any of the four SNPs based on a multiplicative genetic model. Overall there was no significant difference in haplotype frequencies between cases and controls; however, the prevalence of the most common haplotype (ATGC) was significantly higher among cases (38.7%) compared to controls (33.5%) (haplotype-specific *P*=0.009). Evaluation of the prostate cancer risk associated with carrying the ‘ATGC’ haplotype revealed that homozygous carriers were at significantly increased risk (odds ratio (OR)=1.6, 95% confidence interval (CI)=1.2–2.2), compared to noncarriers, while no significant association was found among subjects heterozygous for the haplotype (OR=1.0, 95% CI=0.8–1.2). Restricting analyses to advanced prostate cancer strengthened the association between the ‘ATGC’ haplotype and disease risk (OR for homozygous carriers *vs* noncarriers 1.8, 95% CI=1.3–2.5). In conclusion, the results from this study support the hypothesis that inflammation has a role of in the development of prostate cancer, but further studies are needed to identify the causal variants in this region and to elucidate the biological mechanism for this association.

Chronic inflammatory conditions may increase the incidence of several malignant diseases, including prostate cancer. This association is most well established in individuals with inflammatory bowel disease, but the evidence is strong also for cancers of the bladder, stomach, oesophagus, pancreas and lung (Shacter and Weitzman, 2002). In 1999, De Marzo *et al* (1999) suggested that proliferative inflammatory atrophy may progress to prostatic intraepithelial neoplasia and prostate cancer. In addition, several retrospective case–control studies have observed an association between clinical prostatitis and prostate cancer (Dennis *et al*, 2002). Finally, a systematic genetic analysis revealed an association between prostate cancer risk and sequence variants in *MIC-1, TLR-4* and the *TLR1-TLR6-TLR10* gene cluster, all genes that are important in the inflammatory response (Lindmark *et al*, 2004; Zheng *et al*, 2004; Sun *et al*, 2005).

The interleukin-1 receptor antagonist (IL-1RN) belongs to the IL-1 family that apart from IL-1RN consists of two agonists IL-1*α* and IL-1*β*, and two different receptors, IL-1R type I and IL-1R type II. The *IL-1RN* gene is located on 2q14.2 in close proximity to the genes coding for *IL-1α* and *IL-1β* (Steinkasserer *et al*, 1992). All three molecules bind to the same IL-1 receptors. IL-1*α* and IL-1*β* are proinflammatory cytokines. By binding to the receptors, a cascade of events are initiated leading to recruitment and activation of macrophages and neutrophils, vascular dilation and fever, and a potent proinflammatory immune response (Dinarello, 1988). When the anti-inflammatory cytokine, IL-1RN, binds to the same receptors no signal transduction will be elicited on and thereby the activity of IL-1 is blocked. The relative levels of IL-1RN and IL-1 at an inflammatory site will determine whether a proinflammatory state will be initiated and persist or will be terminated (McIntyre *et al*, 1991).

The IL-1 system plays an important role in protection against many different injuries, ranging from microbial colonisation to malignant transformation (Witkin *et al*, 2002). Relatedly, sequence variants within the *IL-1RN* gene have been associated with various malignancies and infectious diseases (Clay *et al*, 1994; Blakemore *et al*, 1996; El-Omar *et al*, 2000; Machado *et al*, 2001), but the role of IL-1RN polymorphisms in prostate cancer pathogenesis has not been examined.

The aim of this study was to test for association of prostate cancer with sequence variants in the *IL-1RN* gene in a large population-based study in Sweden and in that way evaluate whether polymorphisms in a gene that regulates inflammatory processes might influence the risk of prostate cancer.

## SUBJECTS AND METHODS

### Study population

A detailed description of the study sample was presented elsewhere (Lindmark *et al*, 2004). Briefly, the participants studied come from a large-scale, population-based case–control study in Sweden, CAPS (CAncer Prostate in Sweden). The study population consisted of 1383 prostate cancer case patients and 779 control subjects. The case participants were recruited from four of the six regional cancer registries that cover the entire population of Sweden. Each of these registries serves one health care region (Northern, Central, Stockholm, and South Eastern) and altogether encompasses approximately six million inhabitants (67% of Sweden's population). The cases were linked to the National Prostate Cancer Registry and clinical information such as TNM (tumour-node-metastasis) stage, Gleason sum, PSA level at the time of diagnosis, methods of diagnosis and primary treatment were obtained for 95.3% of the cases. The cases were thereafter classified as either Localised: T1–2 and N0/NX and M0/MX and Grade I–II/Gleason sum 2–7, and PSA<100 or Advanced (having or prone to progressive disease): T3/4 or N+ or M+ or Grade III or Gleason sum 8–10 or PSA>100. For cases with at least one reported family member with prostate cancer, a more detailed family history of prostate cancer was obtained through additional questionnaires and record linkage to Swedish Cancer Registry or medical records. The families were subsequently classified as hereditary prostate cancer: Three or more relatives with prostate cancer (52 cases) or familial prostate cancer: two relatives with prostate cancer (130 cases).

Control subjects were randomly selected from the continuously updated Swedish Population Registry, frequency matched according to the expected age distribution (within 5 years), geographic origin of the cases and sex. Mean age (age at diagnosis for case patients and age at inclusion for control subjects) for the cases and controls were 66.6 and 67.9 years, respectively.

Written informed consent was obtained from each subject. The study was approved by the Ethical Committee at the two participating academic institutions, Karolinska Institutet and Umeå University.

### Genotyping

To test for genetic association, a haplotype tagging single-nucleotide polymorphism (SNPs) approach in the selection of sequence variants was used. First, we selected a subset of common SNPs in the target genomic region of *IL-1RN* using the criteria of SNPs with minor allele frequencies ⩾5% at a density of 1 SNP per kb, with additional attention paid to potentially functional SNPs. In total, 16 SNPs and two repeats (86 bp VNTR and 8 bp repeat) were identified through public databases (NCBI dbSNP and SNPper) (Table 1). The target genomic region was defined as 2 kb of the promoter, all exons, introns, and 3′UTR, for *IL-1RN* this region is approximately 19 kb.

We used three methods for genotyping. First, the 16 identified SNPs and one repeat were genotyped in 94 control subjects using a 5′ nuclease assay with TaqMan MGB probes. The SNP genotyping assays were designed using the Assay-by-Design service, Applied Biosystems (Applied Biosystems Inc., Foster City, CA, USA). The samples were analysed on an ABI 7700 sequence detection system. The VNTR in intron 2 was genotyped using a temperature modulated heteroduplex analysis on a denaturing high-performance liquid chromatography system. This method was performed as described elsewhere (Jonsson *et al*, 2002).

Haplotypes of these SNPs were estimated using a Markov Chain Monte Carlo approach as implemented in the PHASE software package (http://www.stats.ox.ac.uk/mathgen/software.html). Haplotype tagging SNPs (htSNPs), which captured at least 95% of the haplotype variation among the 94 controls, were selected using the htSNP2 computer program (www-gene.cimr.cam.ac.uk/clayton/software/stata). Four htSNPs were identified and they were genotyped in all 1383 cases and 779 controls. The htSNPs (rs878972, rs315934, rs3087263, rs315951) were then genotyped using the MassARRAY system (SEQUENOM, Inc. Valencia, CA, USA). The DNA samples were labelled blindly and shipped from Umeå University, Sweden to the core genotyping laboratory in the Center for Human Genomics, Wake Forest University. In total, 37 controls from the CEPH foundation (1331-01, 1331-02), 29 blind repeats were spread among the DNA samples. In addition, every DNA plate contained two water blanks. A full list of PCR primers and probes and PCR conditions for all systems is available at the author's website (www.wfubmc.edu/genomics).

### Statistical analysis

Hardy–Weinberg equilibrium (HWE) tests for each sequence variant and pairwise linkage disequilibrium (LD) tests for all sequence variants were performed using a replication method, as previously described (Weir, 1996). For each test, 10 000 permutations were performed and the Fisher probability test statistic of each replicate was calculated from the new corresponding multilocus table using the Genetic Data Analysis software package.

Associations between genotypes and prostate cancer were assessed by the score test in conditional logistic regression of a covariate equal to number of rare alleles (0, 1, 2). Genotype-specific risks were estimated as odds ratios (ORs) with associated 95% confidence intervals (CI) by conditional logistic regression. When testing for association and estimating ORs, the conditional logistic regression was stratified by each combination of age (5-year age groups) and geographical region (two regions, as described in the Study Population section) to adjust for the matching conducted in collecting control subjects. Tests for association between haplotypes and prostate cancer risk were performed using a score test developed by Schaid *et al* (2002), using the HAPLO.STAT program (http://www.mayo.edu/hsr/Sfunc.html) for the R programming language. This method, based on the generalised linear model framework, allows adjustment for possible confounding variables and provides both global tests and haplotype-specific tests. In these analyses, age and geographic region were adjusted for through indicator variables representing each combination of age category (5-year age groups) and geographical region (northern part of Sweden *vs* south-eastern part of Sweden and the area of Stockholm). Haplotypes with estimated frequencies less than 0.005 were pooled into a single group. Empirical *P*-values, based on 10 000 simulations, were computed for the global score test and each of the haplotype-specific score tests. All reported *P*-values are two-sided.

## RESULTS

Except for the SNP rs315951, which significantly deviated from HWE in cases (*P*=0.04) but not in controls (*P*=0.34), the remaining three SNPs were consistent with HWE (all *P*>0.05).

No indication of genotyping error was observed. Genotype consistency was 100% among the CEPH control DNA, and results from duplicated samples were 100% concordant, giving an estimated error rate of 0%.

No significant difference in genotype frequencies between cases and controls were observed for any of the four SNPs based on a multiplicative genetic model (Table 2). Assuming a dominant or recessive allelic effect on prostate cancer risk did not alter these findings (data not shown). Stratified analyses based on age (<65 or ⩾65), tumour stage (localised or locally advanced) and family history (sporadic or familial/hereditary) provided no significant differences in genotype frequency between cases and controls (data not shown).

In contrast to comparison of genotypes between cases and controls, an examination of haplotypes yielded evidence of an association with prostate cancer risk. Analysis of the four htSNPs revealed eight major haplotypes, including ATGC, ATGG, CTGC, ACGG, CTAC, ACGC, CTGG, and CTAG of respective SNP rs878972, rs315934, rs3087263, and rs315951. The distribution of these haplotypes was not significantly different between cases and controls (*P*=0.116). However, the prevalence of the most common haplotype (ATGC) was significantly higher among cases (38.7%) compared to controls (33.5%) (haplotype-specific *P*=0.009) (Table 3). Furthermore, in the stratified analysis the frequency of the ‘ATGC’ haplotype was higher in sporadic (39.3%) than in familial (34.8%) prostate cancer cases. Likewise, cases with advanced disease had a higher prevalence (40.0%) than patients with localised disease (37.5%). In addition, we also estimated the prostate cancer risk associated with the most common haplotype. A significantly increased risk was found among homozygous haplotype carriers (OR=1.6, 95% CI=1.2–2.2), compared to noncarriers, while no indication of increased risk was observed among heterozygous carriers (OR=1.0, 95% CI=0.8–1.2). Restricting analyses to aggressive prostate cancer, the risk associated with being homozygous haplotype carrier was further evaluated (OR=1.8, 95% CI=1.3–2.5).

## DISCUSSION

We tested the hypothesis that sequence variants in the *IL-1RN* gene are associated with prostate cancer risk in a large population-based case–control study in Sweden. In this first study of the possible role of *IL1-RN* in prostate cancer aetiology, we found an association with the most common haplotype in *IL1-RN.* Our data are in concordance with the growing evidence that chronic inflammation may play a role in the development of prostate cancer.

The analyses of individual haplotypes showed that the most common haplotype (ATGC) was significantly associated with prostate cancer risk for patients with prostate cancer, and further this association appears to be stronger in cases with advanced disease. The fact that the association was strengthened in cases with advanced disease is of particular note. Genetic markers capable of predicting increased risk of aggressive disease compared with increased risk of any prostate cancer diagnosis are urgently needed, due to the fact that predicting the outcome of prostate cancer is at present very difficult.

The observation that the most common haplotype was associated with prostate cancer risk but none individual htSNPs is of special interest. This highlights the advantages of studying all variation in a gene with the use of a haplotype-tagging approach instead of testing a limited number of single variants. Two different scenarios can explain this observation. First, the association can be a result of two or more sequence variants in the region that individually do not confer a detectably increased risk for prostate cancer, but when using the haplotype-association strategy the impact of the combined risk becomes statistically significant. Second and probably more likely, the ATGC-haplotype may be in strong LD with a risk conferring sequence variant in *IL1-RN*, or in the close vicinity to the gene, while the degree of LD between this risk conferring sequence variant and each of the four htSNPs is of a much weaker magnitude.

The *IL-1RN* gene located on chromosome 2 belongs to the *IL-1* gene cluster spanning over a region of 360-kb (Steinkasserer *et al*, 1992). This cluster of genes contains several pro- and anti-inflammatory cytokine genes that are expressed in both physiological and pathological conditions and plays a key role in the inflammatory immune response. The high density of *IL-1* genes in this region, which share similar functional characteristics, raises the question if an association between a haplotype in *IL-1RN* and prostate cancer, in fact is an association between another gene or genes in the cluster and prostate cancer. Knowledge of the degree of LD across this region is vital to our understanding of which combinations of genotypes are important in disease. Several studies have performed analyses to determine the degree of LD across this region. Consistently, they all show moderate LD in this gene cluster which is not strictly correlated with distances between markers (Cox *et al*, 1998; Bensen *et al*, 2003). To fully answer this question, additional SNPs over this region need to be selected for genetic studies.

The relative levels of IL-1RN together with IL-1*α*/*β* at the site of inflammation will determine whether a proinflammatory reaction will be initiated and persist or will be terminated. An imbalance in pro- and anti-inflammatory cytokines caused by a specific haplotype combination may prevent the normal self-limiting nature of an immune response. A sequence variant in the promoter region of the anti-inflammatory cytokine gene *IL-10* resulted in decreased levels of IL-10 in cases affected with prostate cancer. Further, the decreased level of IL-10 was significant associated with prostate cancer (McCarron *et al*, 2002). Moreover, we recently reported an association between a SNP in the anti-inflammatory cytokine *MIC-1* and prostate cancer (Lindmark *et al*, 2004). MIC-1 is a member of the transforming growth factor *β* superfamily: proteins that play a critical role in the pro- and anti-inflammatory response to infection (Bootcov *et al*, 1997).

Our study has several strengths, which make it informative for studies of the genetic epidemiology of prostate cancer. Firstly, it is large, with DNA samples being available from over 1380 well clinically characterised cases of prostate cancer, and 779 control men known not to have been diagnosed with prostate cancer. Secondly, the ethnic homogeneity of the Swedish population and the ascertainment of age and residence matched control subjects makes us confident that population stratification is not an issue. Finally, the full clinical spectrum of prostate cancer is well represented, with over 40% of the cases having advanced disease.

Test for HWE revealed that the genotype frequencies of one htSNP (rs315951) deviated significantly from expected proportions among prostate cancer subjects. This departure can result from genotyping errors, population stratification, selection, or statistical fluctuations. Considering the high quality in genotyping results (all replicated samples provided concordant genotypes) and the ethnic homogeneity of the Swedish population, it seems most plausible that chance alone is responsible for the observed departure from HWE. Since the departure from HWE was in the direction of excessive homozygosity, the error in haplotype estimations (based on the EM algorithm) is not increased (Fallin and Schork, 2000). Moreover, all statistical inference on haplotypes was based on simulated *P*-values, which are expected to be more robust against departure from HWE compared to the asymptotic distribution of the score statistic.

In summary, we found evidence of an association between a common haplotype in the *IL1RN* gene and prostate cancer in a Swedish population. This finding provides additional support for a role of chronic inflammation in prostate cancer development. Further studies are needed to replicate our finding in other populations and to elucidate the biological function of the *IL1RN* haplotype in relation to prostate cancer risk.

## Figures and Tables

**Table 1:** SNPs selected for genotyping in 94 CAPS control subjects

**SNP name, rs-number**	**Gene location^a^ (relative to the ATG, A=+1)**
IL1RN1129	−1129 C/T
rs2234679	−12 G/C
rs315919	618 C/A
rs4251984	1631 A/G
rs878972	2118 A/C
rs3213448	3702 G/A
IL1RN4696	4696 G/C
rs315936	5352 C/T
rs4251999	6430 −/C
rs315934	8111 G/A
rs392503	8600 T/C
rs3087263	10173 G/A
rs1794068	10908 G/A
rs408392	11863 G/T
VNTRintron2^b^	12560 +/−
rs432014	12985 T/C
rs440286	13874 G/T
rs315951	14992 G/C

a Positions are based on the initiation codon (ATG) from *IL-1RN* genomic DNA (U65590).

b Variable number of an 86-bp tandem repeat present in intron2. Number of copies varies from two to five repeats.

**Table 2:** Genotype frequencies of *IL-1RN* htSNPs in prostate cancer patients and unaffected control subjects

		**Genotype frequencies, # (%)**		
**SNP (position^a^)**	**Genotype**	**Control subjects (*n*=780)**	**Case subjects (*n*=1383)**	***P*-value^b^**
rs878972 (2118A/C)	AA	378 (51.9)	712 (53.4)	
	AC	293 (40.2)	537 (40.3)	
	CC	57 (7.7)	85 (6.4)	0.382

rs315934 (8111G/A)	TT	499 (65.2)	885 (65.8)	
	TC	227 (29.7)	405 (30.1)	
	CC	39 (5.1)	54 (4.0)	0.277

rs3087263 (10173G/A)	GG	632 (81.5)	1144 (83.4)	
	GA	138 (17.8)	218 (15.9)	
	AA	5 (0.6)	10 (0.7)	0.336

rs315951 (14992G/C)	CC	341 (44.2)	640 (46.8)	
	GC	335 (43.4)	566 (41.4)	
	GG	96 (12.4)	162 (11.8)	0.610

a Positions are based on the initiation codon (ATG) from *IL-1RN* genomic DNA (U65590).

b Armitage test for trend on allele counts.

**Table 3:** Estimated haplotype frequencies in controls, patients with sporadic prostate cancer (SPC) and patients with advanced prostate cancer

**Haplotype**				**Controls**	**Cases**			**SPC**			**Advanced PC**		
**rs878972**	**rs315934**	**rs3087263**	**rs315951**	**%**	**%**	**Score^a^**	***P* b**	**%**	**Score^a^**	***P* b**	**%**	**Score^a^**	***P* b**
A	T	G	C	33.5	38.7	2.60	0.009	39.3	2.85	0.006	40.0	2.67	0.009
A	T	G	G	17.6	15.5	−1.01	0.301	15.0	−1.24	0.215	15.0	−1.15	0.246
C	T	G	C	17.5	15.3	−1.16	0.234	15.2	−1.22	0.208	14.1	−1.78	0.080
A	C	G	G	14.3	13.6	−0.58	0.560	14.0	−0.41	0.667	14.9	0.45	0.655
C	T	A	C	8.4	7.7	−0.80	0.428	7.9	−0.64	0.513	8.4	−0.20	0.839
A	C	G	C	5.8	5.4	−0.38	0.713	4.9	−0.89	0.367	4.8	−0.86	0.401
C	T	G	G	1.8	2.7	0.97	0.337	2.9	1.06	0.274	2.4	0.17	0.872
C	T	A	G	0.6	0.7	0.33	0.737	0			0		
Overall							0.116			0.052			0.024

a Score test statistics for association between haplotype and prostate cancer risk.

b Empirical *P*-values based on 10 000 replications.
